# Mixed Effects Modeling Using Stochastic Differential Equations: Illustrated by Pharmacokinetic Data of Nicotinic Acid in Obese Zucker Rats

**DOI:** 10.1208/s12248-015-9718-8

**Published:** 2015-02-19

**Authors:** Jacob Leander, Joachim Almquist, Christine Ahlström, Johan Gabrielsson, Mats Jirstrand

**Affiliations:** 1 Fraunhofer-Chalmers Centre, Chalmers Science Park, SE-41288 Gothenburg, Sweden; 2 Department of Mathematical Sciences, Chalmers University of Technology and University of Gothenburg, Gothenburg, Sweden; 3 Systems and Synthetic Biology, Department of Biology and Biological Engineering, Chalmers University of Technology, Gothenburg, Sweden; 4 CVMD iMed DMPK, AstraZeneca R&D, Mölndal, Sweden; 5 Division of Pharmacology and Toxicology, Department of Biomedical Sciences and Veterinary Public Health, Swedish University of Agricultural Sciences, Uppsala, Sweden

**Keywords:** extended Kalman filter, model uncertainty, nonlinear kinetics, parameter estimation, state prediction

## Abstract

Inclusion of stochastic differential equations in mixed effects models provides means to quantify and distinguish three sources of variability in data. In addition to the two commonly encountered sources, measurement error and interindividual variability, we also consider uncertainty in the dynamical model itself. To this end, we extend the ordinary differential equation setting used in nonlinear mixed effects models to include stochastic differential equations. The approximate population likelihood is derived using the first-order conditional estimation with interaction method and extended Kalman filtering. To illustrate the application of the stochastic differential mixed effects model, two pharmacokinetic models are considered. First, we use a stochastic one-compartmental model with first-order input and nonlinear elimination to generate synthetic data in a simulated study. We show that by using the proposed method, the three sources of variability can be successfully separated. If the stochastic part is neglected, the parameter estimates become biased, and the measurement error variance is significantly overestimated. Second, we consider an extension to a stochastic pharmacokinetic model in a preclinical study of nicotinic acid kinetics in obese Zucker rats. The parameter estimates are compared between a deterministic and a stochastic NiAc disposition model, respectively. Discrepancies between model predictions and observations, previously described as measurement noise only, are now separated into a comparatively lower level of measurement noise and a significant uncertainty in model dynamics. These examples demonstrate that stochastic differential mixed effects models are useful tools for identifying incomplete or inaccurate model dynamics and for reducing potential bias in parameter estimates due to such model deficiencies.

## INTRODUCTION

In pharmacokinetic and pharmacodynamic modeling, the physical system is often assumed to be described by a system of ordinary differential equations (ODEs). In pharmacokinetics, compartmental models are mostly used, whereas for pharmacodynamics, direct or turnover response models are common (1). The observed data are assumed to arise from a deterministic process under some measurement noise (additive, proportional, a combination of the two, or more general probabilistic models). However, the use of deterministic modeling approaches for describing the dynamics often suffers from limited and uncertain knowledge regarding the details of such processes. Since it is up to the modeler to define a model describing the drug administration and its effect, there is also an uncertainty in the model itself. This uncertainty is not explicitly accounted for when considering a deterministic model, for example when using ODEs to describe the dynamics together with a measurement model to incorporate the error. This can lead to model deficiencies, such as correlated residuals, overestimated measurement noise, and incorrect inference (2).

Nonlinear mixed effects (NLME) models were introduced into the pharmaceutical field to analyze data from several individuals simultaneously (3–5). The individuals are assumed to be described by a common structural model with some of the model parameters varying within the population (so-called random effects parameters), while other parameters are invariant between subjects (so-called fixed effects parameters). The NLME approach can be of great benefit when the data is sparse and the information from a single subject is not sufficient to identify the model parameters. It is typically performed using a deterministic model describing the underlying system, for example by utilizing ODEs (6, 7).

We consider the extension of the NLME approach to allow for uncertainty in the model dynamics. This is done by considering stochastic differential equations (SDEs), which is an extension of ODEs to allow for a random part in the model dynamics. This approach has previously been advocated, see for example (8–10). We have previously also demonstrated the benefits of using SDEs when solving the inverse problem of parameter estimation (11). SDEs can furthermore be used to model the inherent randomness in pharmacokinetic and pharmacodynamic systems (12, 13), as an alternative to discrete models using a master equation approach and the Gillespie algorithm (14–16). Also, in systems without true randomness, SDEs can be used to model an incomplete or imperfect model structure. SDEs (see (17, 18) for references) have long been used in mathematical finance, for example to model the uncertainty in an asset (19). In contrast to the classical approach, where data variability arises from the measurements and the variability in parameters, a stochastic model also incorporates errors in the dynamics itself. Hence, this kind of modeling allows for three different sources of variability: population variability, measurement error, and system noise.

One often faces the inverse problem of estimating model parameters from observed noisy data. There are several approaches to the delicate problem of estimating parameters in stochastic differential mixed effects models. In general, there is no closed form solution of the likelihood function. Approaches to the parameter estimation problem on a population level include for example the first-order (FO) and the first-order conditional estimation (FOCE) method (20, 21) and stochastic approximation of expectation maximization (SAEM) method (22). State estimation on an individual level includes, for example, the Kalman filter (KF), the extended Kalman filter (EKF), and particle filters. For a combination of the FOCE approximation of the population likelihood and the EKF, see for example (10, 23–25). In (26), the authors propose a combination of the SAEM algorithm and EKF. For a review of parameter estimation methods in SDE population models, see (27).

In this paper, we consider approximation of the population likelihood by using the first-order conditional estimation with interaction (FOCEI) approximation of the population likelihood together with the EKF for state estimation on the individual level. In contrast to previous efforts (10, 23–25), we estimate the full covariance matrix describing population variability. That is, we allow for correlation between random parameters in the model. By utilizing the FOCEI method, we allow for interaction between output variance and random parameters. Furthermore, instead of adopting the commonly used finite difference approximation, we make use of sensitivity equations to evaluate the gradient of the objective function in the optimization procedure, previously mentioned in (28). Moreover, we produce illustrative plots describing state variable uncertainty and output uncertainty (which is a combination of state and measurement uncertainty). These plots serve as diagnostic tools of model appropriateness and illustration of the uncertainty in model output.

The extension of ODEs to SDEs is illustrated using two examples of pharmacokinetic data. First, a stochastic one-compartmental pharmacokinetic model with first-order input and nonlinear elimination is considered by using a simulated data set consisting of 20 animals. Since we now account for three sources of variability in data, it is important to know if the three sources can be distinguished from each other. From the simulated data, the parameters of the model are estimated, including the system noise and the covariance matrices describing measurement error and parameter variability. Second, we consider a data set from a preclinical study of nicotinic acid (NiAc) turnover in obese rats, where the original NiAc disposition model and a NiAc disposition model extended to an SDE model are compared in terms of parameter estimates and model prediction.

## MATERIALS AND METHODS

### Mathematical Theory

In this section, we state the stochastic mixed effects model and derive the (approximate) maximum likelihood theory needed for parameter estimation. This section is recommended for readers not familiar with the concept of SDEs. We also introduce the concept of the EKF, which serves as a state estimator for the stochastic model (29).

#### The Stochastic Mixed Effects Model Framework

In population modeling, NLME models are used to describe data of the form

1 $$ {\boldsymbol{y}}_{ij},i=1, \dots, N,j=1,\dots, {n}_i, $$

where the vector ***y***_*ij*_ denotes the *j*:th observation for the *i*:th individual. The statistical model is the following

2 $$ d{\boldsymbol{x}}_i=\boldsymbol{f}\left({\boldsymbol{x}}_i,{\boldsymbol{u}}_i,t,{\boldsymbol{\phi}}_i\right)dt,{\boldsymbol{x}}_i(0)={\boldsymbol{x}}_0\left({\boldsymbol{\phi}}_i\right) $$

3 $$ {\boldsymbol{y}}_{ij}=\boldsymbol{h}\left({\boldsymbol{x}}_i,{\boldsymbol{u}}_i,{t}_{ij},{\boldsymbol{\phi}}_i\right)+{\boldsymbol{e}}_{ij}, $$

where time is denoted by *t*. Note that the differential equation is written in differential form, which is the standard notation for SDEs that will be introduced later on. The vector-valued function ***f***(***x***_*i*_, ***u***_*i*_, *t*, ***ϕ***_*i*_) describes the dynamics of the system, and ***ϕ***_*i*_ denotes the individual parameters for individual *i*. The state variables of the system are denoted ***x***_*i*_ and may for example be the concentration of a drug or drug effect. The input to the system is denoted ***u***_*i*_, which for example can be an infusion. Measurements are assumed to be taken at discrete points in time and characterized by the measurement function ***h***(***x***_*i*_, ***u***_*i*_, *t*_*ij*_, ***ϕ***_*i*_) and the measurement error ***e***_*ij*_ ~ *N*(**0**, ***S***(***x***_*i*_, ***u***_*i*_, *t*_*ij*_, ***ϕ***_*i*_)).

The individual parameters ***ϕ***_*i*_ are related to the population parameters ***θ*** according to

$$ {\boldsymbol{\phi}}_i=\boldsymbol{g}\left(\boldsymbol{\theta}, {\boldsymbol{Z}}_i,{\boldsymbol{\eta}}_i\right), $$

where ***θ*** denote the fixed effect parameters, ***Z***_*i*_ denote the covariates for individual i and ***η***_*i*_ ~ N(**0**, **Ω**) are the random effects for individual *i*, which are assumed to be multivariate normal distributed with mean zero and covariance **Ω**. The model described above is the commonly used NLME model setup, and we refer the reader to (6, 7) for more information.

Instead of considering the deterministic model in the classic framework, we want to include some kind of uncertainty in the differential equations as well. This is achieved by expanding the ODEs to SDEs. The stochastic differential mixed effect model, abbreviated SDMEM (30), is defined as

4 $$ d{\boldsymbol{x}}_i=\boldsymbol{f}\left({\boldsymbol{x}}_i,{\boldsymbol{u}}_i,t,{\boldsymbol{\phi}}_i\right)dt+\boldsymbol{\Sigma} \left({\boldsymbol{x}}_i,{\boldsymbol{u}}_i,t,{\boldsymbol{\phi}}_i\right)d{\boldsymbol{W}}_i,\kern0.5em {\boldsymbol{x}}_i(0)={\boldsymbol{x}}_0\left({\boldsymbol{\phi}}_i\right) $$

5 $$ {\boldsymbol{y}}_{ij}=\boldsymbol{h}\left({\boldsymbol{x}}_i,{\boldsymbol{u}}_i,{t}_{ij},{\boldsymbol{\phi}}_i\right)+{\boldsymbol{e}}_{ij}. $$

An SDE of the form (4) consists of two parts. First, we have the so-called drift function ***f***(***x***_*i*_, ***u***_*i*_, *t*, ***ϕ***_*i*_) corresponding to the deterministic part in the model, which is the same as in Eq. (2). Second, we have the random term **Σ**(***x***_*i*_, ***u***_*i*_, *t*, ***ϕ***_*i*_)*d* ***W***_*i*_, which corresponds to the uncertain part of the model. We will later refer to **Σ**(***x***_*i*_, ***u***_*i*_, *t*, ***ϕ***_*i*_)*d* ***W***_*i*_ as the system noise. The system noise is a continuous stochastic process, in contrast to the measurement noise, which is realized at discrete time points. In Eq. (4), *d* ***W***_*i*_ corresponds to the increment of a q-dimensional Wiener process ***W***_*i*_. The elements of *d* ***W***_*i*_ are independent and normally distributed with mean zero and variance *d* *t*. Moreover, the Wiener increments *d* ***W***_*i*_ are considered independent across individuals and independent of the measurement error.

In contrast to the classic approach, where the only error arises in the measurement equation, an SDE setting provides a flexible framework to account for fluctuations in the underlying state variables. The system noise is a tool that accounts for all the unknown phenomena that are not captured by the deterministic model, for example approximations, modeling errors, and oversimplifications. In a mixed effects model setting, variability in response can now arise from three different sources, namely measurement noise, system noise, and parameter variability.

#### Parameter Estimation in the Stochastic Mixed Effects Population Framework

Given a collection of measurements of the form (2) and an underlying model of the form (4)–(5), the model parameters can be estimated using the maximum likelihood approach. This has previously been elaborated, see, e.g., (10, 25). For convenience of the reader, we here provide the necessary equations together with the extension to models with interaction between random effects and output covariance.

For a specific individual *i*, the optimal parameter values are found by maximizing the individual likelihood. Using the notation *Y*_*ik*_ = [***y***_*i*1_, ***y***_*i*2_, …, ***y***_*ik*_] to denote the measurements up to time point *t*_*k*_ for individual *i*, the combined likelihood becomes

$$ {L}_i\left(\boldsymbol{\theta} \Big|{Y}_{i{n}_i}\right)=\left(\prod_{j=2}^{n_i}p\left({\boldsymbol{y}}_{ij}\Big|{Y}_{i\left(j-1\right)},\boldsymbol{\theta} \right)\right)p\left({\boldsymbol{y}}_{i1}\Big|\boldsymbol{\theta} \right) $$

where the probability for an observation given the previous observations and the parameters is *p*(***y***_*ij*_|*Y*_*i*(*j* − 1)_, ***θ***). Assuming Gaussian densities, which are characterized by their first and second moments denoted by

$$ \begin{array}{l}{\widehat{\boldsymbol{y}}}_{ij}=E\left({\boldsymbol{y}}_{ij}\Big|{Y}_{i\left(j-1\right)},\boldsymbol{\theta} \right)\hfill \\ {}{\boldsymbol{R}}_{ij}=Var\left({\boldsymbol{y}}_{ij}\Big|{Y}_{i\left(j-1\right)},\boldsymbol{\theta} \right),\hfill \end{array} $$

we can write down the individual likelihood. Taking the logarithm, the individual log-likelihood is given by

$$ \log {L}_i\left(\boldsymbol{\theta} \Big|{Y}_{i{n}_i}\right)=-\frac{1}{2}{\displaystyle \sum_{j=1}^{n_i}}\left({\boldsymbol{\epsilon}}_{ij}^T{\boldsymbol{R}}_{ij}^{-1}{\boldsymbol{\epsilon}}_{ij}+ \log \left|2\pi {\boldsymbol{R}}_{ij}\right|\right), $$

where

$$ {\boldsymbol{\epsilon}}_{ij}={\boldsymbol{y}}_{ij}-{\hat{\boldsymbol{y}}}_{ij}, $$

is the prediction error, assumed to be normal distributed with mean **0** and variance ***R***_*ij*_. We denote the collection of all individual measurements $Y=\left\{{Y}_{1{n}_1},{Y}_{2{n}_2},\dots, {Y}_{N{n}_N}\right\}$. The population likelihood is simply a product of individual likelihoods,

$$ L\left(\boldsymbol{\theta} \Big|Y\right)=\prod_{i=1}^Np\left({Y}_{i{n}_i}\Big|\boldsymbol{\theta}, \boldsymbol{\Omega} \right) $$

Since the random effects are unobserved quantities, we marginalize over the random effects,

6 $$ L\left(\boldsymbol{\theta} \Big|Y\right)=\prod_{i=1}^N{\displaystyle \int }p\left({Y}_{i{n}_i}\Big|\boldsymbol{\theta}, {\boldsymbol{\eta}}_i\right)p\left({\boldsymbol{\eta}}_i\Big|\boldsymbol{\Omega} \right)d{\boldsymbol{\eta}}_i=\prod_{i=1}^N \exp \left({l}_i\right)d{\boldsymbol{\eta}}_i, $$

where ${l}_i={l}_i\left({\boldsymbol{\eta}}_i\right)={l}_i\left({\boldsymbol{\eta}}_i;{Y}_{i{n}_i},\boldsymbol{\theta} \right)$ is the *a posteriori* log-likelihood for the random effects of the *i*:th individual

7 $$ {l}_i=-\frac{1}{2}{\displaystyle \sum_{j=1}^{n_i}}\left({\boldsymbol{\epsilon}}_{ij}^T{\boldsymbol{R}}_{ij}^{-1}{\boldsymbol{\epsilon}}_{ij}+ \log \left|2\pi {\boldsymbol{R}}_{ij}\right|\right)-\frac{1}{2}{\boldsymbol{\eta}}_i^T{\boldsymbol{\Omega}}^{-1}{\boldsymbol{\eta}}_i-\frac{1}{2} \log \left|2\pi \boldsymbol{\Omega} \right|, $$

In most cases, there is no closed form expression for the integral in Eq. (6). The integral can be approximated using the Laplace approximation, see (31–33). The Laplace approximation uses a second-order Taylor expansion of *l*_*i*_ around a point ***η***_i_^*^. Here, the point is chosen to be the value of ***η***_*i*_ which maximizes the individual log-likelihood (7),

$$ {\boldsymbol{\eta}}_i^{\ast }=\underset{{\boldsymbol{\eta}}_i}{ \arg \kern0.15em  \max}\kern0.28em {l}_i\left({\boldsymbol{\eta}}_i\right). $$

Using this ***η***_i_^*^, we end up with the approximate population likelihood function

$$ L\left(\boldsymbol{\theta} \Big|Y\right)\approx \prod_{i=1}^N \exp \left({l}_i\left({\boldsymbol{\eta}}_i^{\ast}\right)\right)\left|\frac{-\Delta {l}_i\left({\boldsymbol{\eta}}_i^{*}\right)}{2\pi}\right|, $$

where Δ*l*_*i*_(***η***_i_^*^) denotes the Hessian of the individual log-likelihood (7) evaluated at the point ***η***_i_^*^. Taking the logarithm, we have

$$ \log L\left(\boldsymbol{\theta} \Big|Y\right)\approx {\displaystyle \sum_{i=1}^N}{l}_i\left({\boldsymbol{\eta}}_i^{*}\right)-\frac{1}{2} \log \left|\frac{-\Delta {l}_i\left({\boldsymbol{\eta}}_i^{*}\right)}{2\pi}\right|. $$

The expression for the element at index *l*, *k* of the Hessian matrix is

$$ \begin{array}{c}\kern1em {\left(\varDelta {l}_i\left(\boldsymbol{\eta} \right)\right)}_{l,k}=\frac{\partial^2{l}_i\left(\boldsymbol{\eta} \right)}{\partial {\eta}_l\partial {\eta}_k}\kern1em \\ {}\kern1em \approx -{\displaystyle \sum_{j=1}^{n_i}\frac{\partial {\boldsymbol{\epsilon}}_{ij}^T}{\partial {\eta}_l}{\boldsymbol{R}}_{ij}^{-1}\frac{\partial {\boldsymbol{\epsilon}}_{ij}}{\partial {\eta}_k}+{\boldsymbol{\epsilon}}_{ij}^T{\boldsymbol{R}}_{ij}^{-1}\frac{\partial {\boldsymbol{R}}_{ij}}{\partial {\eta}_l}{\boldsymbol{R}}_{ij}^{-1}\frac{\partial {\boldsymbol{\epsilon}}_{ij}^T}{\partial {\eta}_k}+\frac{\partial {\boldsymbol{\epsilon}}_{ij}^T}{\partial {\eta}_k}{\boldsymbol{R}}_{ij}^{-1}{\boldsymbol{\epsilon}}_{ij}}\kern1em \\ {}\kern1em -{\boldsymbol{\epsilon}}_{ij}^T{\boldsymbol{R}}_{ij}^{-1}\frac{\partial {\boldsymbol{R}}_{ij}}{\partial {\eta}_l}{\boldsymbol{R}}_{ij}^{-1}\frac{\partial {\boldsymbol{R}}_{ij}}{\partial {\eta}_k}{\boldsymbol{\epsilon}}_{ij}-\frac{1}{2}Tr\left(-{\boldsymbol{R}}_{ij}^{-1}\frac{\partial {\boldsymbol{R}}_{ij}}{\partial {\eta}_l}{\boldsymbol{R}}_{ij}^{-1}\frac{\partial {\boldsymbol{R}}_{ij}}{\partial {\eta}_k}\right)-{\varOmega}_{l,k}^{-1},\kern1em \end{array} $$

where only first-order partial derivatives are considered and higher-order contributions are assumed to be negligible in the calculation of the Hessian Δ*l*_*i*_(*η*). This is called the FOCEI approximation. If we assume no interaction between the output covariance ***R***_*ij*_ and the random parameters, the approximate Hessian is given by

9 $$ {\left(\Delta {l}_i\left(\boldsymbol{\eta} \right)\right)}_{l,k}=\frac{\partial^2{l}_i\left(\boldsymbol{\eta} \right)}{\partial {\eta}_l\partial {\eta}_k}\approx -{\displaystyle \sum_{j=1}^{n_i}}\frac{\partial {\boldsymbol{\epsilon}}_{ij}^T}{\partial {\eta}_l}{\boldsymbol{R}}_{ij}^{-1}\frac{\partial {\boldsymbol{\epsilon}}_{ij}}{\partial {\eta}_k}-{\Omega}_{l,k}^{-1}, $$

referred to as the FOCE method (33). Finally, the maximum likelihood estimates are given by maximizing the approximate population likelihood as

10 $$ \widehat{\boldsymbol{\theta}}=\underset{\boldsymbol{\theta}}{ \arg \kern0.15em  \max } \log \kern0.15em L\left(\boldsymbol{\theta} \Big|Y\right). $$

Due to the stochastic part of the model in Eqs. (4)–(5), the state of the system is uncertain. There are several solutions to the state estimation problem in these situations, including for example particle filters and the Kalman filter (29). In this paper, we will utilize the so-called extended Kalman filter (EKF).

#### The Continuous Discrete Extended Kalman Filter

To calculate the individual log-likelihoods (7), we need the prediction errors ***ϵ***_*ij*_ and the output covariance matrices ***R***_*ij*_. As noted in (10, 25), these identities can be recursively computed using the extended Kalman filter (EKF).

The continuous discrete EKF is a state estimator for continuous discrete state space models of the form (4)–(5) (29). From observations, the state variables of the system and their covariance are estimated in order to compute the residuals and the output covariance. From now on, we drop the individual notation *i*.

The EKF is an extension of the famous Kalman filter to nonlinear models (34). For linear dynamic models, the Kalman filter provides an optimal state estimator for a given parameter vector ***ϕ***. For nonlinear models, the EKF uses a first-order linearization around the model trajectory. The EKF provides estimates of the conditional expectation of the state ${\hat{\boldsymbol{x}}}_{k|k}=E\left({\boldsymbol{x}}_{t_k}\Big|{Y}_k,\boldsymbol{\phi} \right)$ and its covariance ${\boldsymbol{P}}_{k|k}=Var\left({\boldsymbol{x}}_{t_k}\Big|{Y}_k,\boldsymbol{\phi} \right)$. Given initial conditions ${\hat{\boldsymbol{x}}}_{1|0}={\boldsymbol{x}}_0$ and ***P***_1|0_ = ***P***_0_ and linearizations

$$ \begin{array}{l}{\boldsymbol{A}}_t=\frac{\partial \boldsymbol{f}}{\partial {\boldsymbol{x}}_t}\Big|{}_{{\boldsymbol{x}}_t={\widehat{\boldsymbol{x}}}_{t|k}}\kern1em \\ {}{\boldsymbol{C}}_k=\frac{\partial \boldsymbol{h}}{\partial {\boldsymbol{x}}_t}\Big|{}_{{\boldsymbol{x}}_t={\widehat{\boldsymbol{x}}}_{k|k-1}},\kern1em \end{array} $$

the state variables and their covariance are predicted between two consecutive measurement time points according to

$$ \begin{array}{l}\frac{d{\widehat{\boldsymbol{x}}}_{t|k}}{dt}=\boldsymbol{f}\left({\widehat{\boldsymbol{x}}}_{t|k},{\boldsymbol{u}}_t,t,\boldsymbol{\phi} \right),t\in \left[{t}_k,{t}_{k+1}\right]\kern1em \\ {}\frac{d{\boldsymbol{P}}_{t|k}}{dt}={\boldsymbol{A}}_t{\boldsymbol{P}}_{t|k}+{\boldsymbol{P}}_{t|k}{\boldsymbol{A}}_t+\boldsymbol{\Sigma} {\boldsymbol{\Sigma}}^T,t\in \left[{t}_k,{t}_{k+1}\right].\kern1em \end{array} $$

From the predicted state variables and their covariance, we have the output prediction equations

$$ \begin{array}{l}{\widehat{\boldsymbol{y}}}_{k|k-1}=\boldsymbol{h}\left({\widehat{\boldsymbol{x}}}_{k|k-1},{\boldsymbol{u}}_k,{t}_k,\boldsymbol{\phi} \right)\kern1em \\ {}{\boldsymbol{R}}_{k|k-1}={\boldsymbol{C}}_k{\boldsymbol{P}}_{k|k-1}{\boldsymbol{C}}_k^T+\boldsymbol{S}.\kern1em \end{array} $$

From the state covariance ***P***_*k*|*k* − 1_ and measurement covariance ***R***_*k*|*k* − 1_, the Kalman gain is given by

$$ {\boldsymbol{K}}_k={\boldsymbol{P}}_{k|k-1}{\boldsymbol{C}}_k^T{\boldsymbol{R}}_{k|k-1}^{-1}. $$

Finally, the state and its covariance are updated according to

$$ \begin{array}{l}{\widehat{\boldsymbol{x}}}_{k|k}={\widehat{\boldsymbol{x}}}_{k|k-1}+{\boldsymbol{K}}_k{\boldsymbol{\epsilon}}_k\kern1em \\ {}{\boldsymbol{P}}_{k|k}={\boldsymbol{P}}_{k|k-1}-{\boldsymbol{K}}_k{\boldsymbol{R}}_{k|k-1}{\boldsymbol{K}}_k^T,\kern1em \end{array} $$

where the residual ***ϵ***_*k*_ is given by

$$ {\boldsymbol{\epsilon}}_k={\boldsymbol{y}}_k-{\widehat{\boldsymbol{y}}}_{k|k-1}. $$

#### Optimization of the Approximate Population Likelihood

To maximize the approximate population likelihood in Eq. (10), we have to solve a nested optimization problem. For every value of the population parameters ***θ*** in the optimization of the approximate population likelihood, the individual likelihoods in Eq. (7) have to be maximized with respect to the random effects due to the Laplace approximation. We refer to the maximization of the individual likelihoods as the inner optimization problem and maximization of the approximate population likelihood as the outer optimization problem.

For the outer and inner optimization problems, we use a local gradient-based quasi-Newton optimization routine based on the Broyden-Fletcher-Goldfarb-Shannon (BFGS) updating formula (35). The BFGS updating formula is a popular optimization method because it performs well in many different problems.

Since the optimization methods are gradient-based, we need to calculate the gradient of the outer objective function (10) and the gradient of the inner objective function (7). We also need to calculate the approximate Hessians (8) or (9) of the inner objective function. As argued in (28), there are three approaches to this problem, namely (i) approximations based on finite differences, (ii) symbolic differentiation, or (iii) automatic differentiation tools. Instead of using finite difference approximation, as performed in (8, 10), we use symbolic differentiation using the symbolic algebra capability in Mathematica. By using symbolic derivation, the system of ODEs if differentiated with respect to the model parameters to obtain the so-called sensitivity equations. These sensitivity equations are integrated together with the original system of ODEs to form the expression of the gradient.

- Application 1: Simulation and estimation of a stochastic one-compartmental pharmacokinetic model

To illustrate the concept of SDEs explained in the previous section, we consider a one-compartment pharmacokinetic model with first-order input and nonlinear elimination. In this application, we validate the proposed stochastic modeling framework. A necessary condition is that the model parameters and the three sources of variability (measurement error, population variability, and model uncertainty) can be identified. Here, we consider data from a simulated population consisting of 20 animals. Moreover, we are interested in investigating the difference in parameter estimates with an assumption of a deterministic model. Consider the deterministic mixed effects pharmacokinetic model

$$ \begin{array}{ll}\frac{d{A}_i}{dt}=-{k}_{ai}{A}_i,\hfill & {A}_i(0)=20\hfill \\ {}V\frac{d{C}_i}{dt}={k}_{ai}{A}_i-\frac{V_{mi}}{K_m+{C}_i}{C}_i,\hfill & {C}_i(0)=0,\hfill \end{array} $$

where *A*_*i*_ (mg) and *C*_*i*_ (mg L^−1^) are the amount of drug in the GI tract and the concentration of drug in plasma for individual *i*, respectively. We assume that the *k*_*ai*_ and *V*_*mi*_ are multivariate log-normal distributed, that is

$$ \begin{array}{l}{k}_{ai}={k}_a \exp \left({\eta}_{i1}\right),\hfill \\ {}{V}_{mi}={V}_m \exp \left({\eta}_{i2}\right),\hfill \end{array} $$

where the random effects vector ***η*** = (*η*_*i*1_, *η*_*i*2_) is assumed to follow a multivariate normal distribution with mean zero and covariance matrix **Ω**. The parameters of the model are the *k*_*a*_ (min^−1^), *V*_*m*_ (mg min^−1^), *K*_*m*_ (mg L^−1^), and *V* (L). Moreover, we parameterize **Ω** = ***UU*** ^*T*^, where ***U*** is an upper triangular matrix of the form

$$ \boldsymbol{U} = \left(\begin{array}{cc}\hfill {\omega}_{11}\hfill & \hfill {\omega}_{12}\hfill \\ {}\hfill 0\hfill & \hfill {\omega}_{22}\hfill \end{array}\right). $$

The reason for the parameterization **Ω** = ***UU*** ^*T*^ is to assure that **Ω** is a positive definite matrix, a necessary condition since it is a covariance matrix. We get

$$ \boldsymbol{\Omega} = \left(\begin{array}{cc}\hfill {\omega}_{11}^2+{\omega}_{12}^2\hfill & \hfill {\omega}_{12}{\omega}_{22}\hfill \\ {}\hfill {\omega}_{12}{\omega}_{22}\hfill & \hfill {\omega}_{22}^2\hfill \end{array}\right). $$

Using additive system noise, we end up with an SDE describing the concentration of drug.

11 $$ Vd{C}_i=\left({k}_{ai}{A}_i-\frac{V_{mi}}{K_m+{C}_i}{C}_i\right)dt+\sigma d{W}_i,\ {C}_i(0)=0. $$

In the stochastic model, there exists a system noise *σd* ***W***_*i*_, where *σ* is the scaling factor and *d* ***W***_*i*_ is the increment of a standard Wiener process. In Eq. (11), the system noise is independent of the drug concentration. This may not be a realistic assumption since it allows for a change in concentration even in the absence of drug. By defining the system noise dependent on the concentration level itself, such phenomena can be avoided. The final model, which we consider for simulation and estimation, is

12 $$ \begin{array}{cc}\hfill \frac{d{A}_i}{dt} = -{k}_{ai}{A}_i,\hfill & \hfill {A}_i(0)=20\hfill \end{array} $$

13 $$ \begin{array}{cc}\hfill Vd{C}_i=\left({k}_{ai}{A}_i-\frac{V_{mi}}{K_m+{C}_i}{C}_i\right)dt+\sigma {\hat{C}}_id{W}_i,\hfill & \hfill {C}_i(0)=0,\hfill \end{array} $$

where *σ* (L min^−1^) is the system noise factor. In the SDE above, *Ĉ*_*i*_ denotes the approximation of the conditional expectation of the concentration *C*_*i*_ computed by the EKF. The reason for this model is that the EKF does not allow for state-dependent system noise. However, by utilizing the approximation of the conditional expectation of the concentration, we can still use a proportional-like system noise. For SDEs with system noise dependent on the stochastic process itself, one can in rare cases use the Lamperti transform to obtain a state-independent system noise. The SDE (12)–(13) has a stochastic part in the equation describing the central compartment. Since *dW*_*i*_ is the increment of a Wiener process, this implies that *dW*_*i*_ is normally distributed with variance *dt*, which in turn implies that *σĈ*_*i*_ *dW*_*i*_ is normally distributed with variance *σ* ^2^ *Ĉ*_*i*_^2^ *dt*.

Mass-balance constrains for SDE models can be enforced by assign the structure of **Σ** according to the stoichiometry of the modeled system. For example, accounting for uncertainty in the drug uptake rate from the GI tract can be achieved by adding the same system noise term to the Eq. (12) with opposite sign. However, we have chosen to assume that the system noise describes an uncertainty in the elimination process from the central compartment, requiring no mass-balancing.

Furthermore, the measurement *y*_*ik*_ is the concentration for individual *i* measured at time *t*_*ik*_ under additive Gaussian noise according to

14 $$ {y}_{ik}={C}_i\left({t}_{ik}\right)+{e}_{ik}, $$

where *e*_*ik*_ ~ *N*(0, *s* ^2^). A population consisting of 20 animals is simulated according to the parameter values in the third column of Table I. A total of 100 data sets are simulated, and the model parameters are estimated using the FOCEI method. The response is measured at equidistant time points *t*_*k*_ = 1, 9, 17, …, 97 minutes for all animals.

**Table I:** Estimated parameter values for the one-compartmental model (12)–(13) using the ODE and the SDE model

Parameter	Definition	True value	Starting value	ODE model (RSE %)	SDE model (RSE %)
*k*_*a*_	First-order absorption	0.1	0.2	0.078 (22.6)	0.103 (12.9)
*V*_*m*_	Maximal velocity	0.5	1	0.637 (20.3)	0.508 (9.52)
*K*_*m*_	Michaelis-Menten const.	3	1	5.43 (51.3)	3.04 (14.9)
*V*	Compartmental volume	1	2	0.793 (17.6)	1.00 (5.50)
*s*	Measurement error std.	0.1	0.5	0.609 (17.2)	0.099 (7.49)
*ω*_11_	Interindividual variability *k*_*a*_	0.5	0.1	0.403 (22.8)	0.456 (18.4)
*ω*_12_	Interindividual correlation	0.1	0	0.098 (120.9)	0.092 (149.5)
*ω*_22_	Interindividual variability *V*_*m*_	0.3	0.1	0.332 (18.3)	0.279 (18.9)
*σ*	System noise factor	0.05	0.01	-	0.050 (6.56)

The relative standard errors (RSEs) in % are included in parenthesis

- Application 2: Stochastic NiAc disposition in obese Zucker rats

In this section, we extend a pharmacokinetic model of nicotinic acid (NiAc) in obese Zucker rats, previously used to drive a pharmacodynamic model describing nonesterified fatty acid (NEFA) turnover (36–41). The disposition of NiAc in obese rats was described by a one-compartment model with endogenous synthesis of NiAc and a capacity-limited elimination process. Instead of using a deterministic model for the pharmacokinetics, we consider the extension to a stochastic NiAc disposition model.

#### Original NiAc Disposition Model

The previously used pharmacokinetic model was a one-compartmental model with a synthesis rate *Synt* ($\mu$mol min^−1^ kg^−1^) of NiAc in the absence of drug with a nonlinear elimination with parameters *V*_*mi*_ ($\mu$mol min^−1^ kg^−1^) and *K*_*m*_ ($\mu$mol L^−1^) describing the maximal rate from the central compartment and the Michaelis-Menten constant, respectively. The mixed effects kinetics is described by the ODE

15 $$ {V}_c\frac{d{c}_i}{dt}=u+ Synt-\frac{V_{mi}{c}_i}{K_m+{c}_i}, $$

16 $$ {c}_i(0)=\frac{Synt\ {K}_m}{V_{mi}- Synt}, $$

where *u* ($\mu$mol min^−1^ kg^−1^) denotes the input. Two different infusion rates were used, 0.67 $\mu$mol min^−1^ kg^−1^ (corresponding to 20 $\mu$mol kg^−1^ over 30 min) and 0.17 $\mu$mol min^−1^ kg^−1^ (corresponding to 51 $\mu$mol kg^−1^ over 300 min).

In previous work, in addition to *V*_*mi*_, *Synt* was allowed to be distributed in the population. In this paper, we only consider *V*_*mi*_, to be distributed in the population. This choice was due to the fact that few data samples were taken at steady-state and that the estimation results in (39) showed a very high residual standard error on the interindividual variability parameters. We consider a log-normal distribution of the maximal rate *V*_*mi*_. That is

$$ {V}_{mi}={V}_m \exp \left({\eta}_i\right), $$

where *η*_*i*_ ~ *N*(0, *ω* ^2^). *V*_*c*_ (L kg^−1^) denotes the central volume. The first group consisted of eight subjects and the second group of seven subjects. There was also one subject receiving placebo, giving a total of 16 subjects in the analysis.

#### Stochastic NiAc Disposition Model

We consider the extension to a stochastic NiAc model described by the SDE

17 $$ {V}_cd{c}_i=\left(u+ Synt-\frac{V_{mi}{c}_i}{K_m+{c}_i}\right)dt+\sigma {\widehat{c}}_id{W}_i $$

In the stochastic NiAc disposition model, the system noise *σĉ*_*i*_ *dW*_*i*_ models the uncertainty in dynamics. Again, we assume a system noise proportional to the mode *ĉ*_*i*_. We refer to *σ* (L min^−1^ kg^−1^) as the system noise factor. In contrast to the original NiAc disposition model, the total error is now divided into measurement error and system noise. The choice of the stochastic model structure is to allow for an uncertainty in the elimination process dependent on the drug concentration.

The purpose of the extension is to identify the structural parameters together with the sources of variability. Most importantly, we are interested in identifying the system noise factor *σ*. Note that *σ* = 0 corresponds to the original NiAc disposition model.

## RESULTS

- Application 1: Simulation and estimation of a stochastic one-compartmental pharmacokinetic model

In this example, the primary interest lies in how well the parameters in the model can be estimated from data. Since we have three sources of variability (parameter variability, measurement error, and system noise), it is important to know whether these sources of variability can be separated in the estimation.

The parameters in the model are estimated using the FOCEI method. The parameters in the model consist of the structural parameters *k*_*a*_, *V*_*m*_, *K*_*m*_ and *V* and the parameters *s*, *ω*_11_, *ω*_12_, *ω*_22_ and *σ* describing the three sources of variability. Hence, our vector of model parameters is ***θ*** = (*k*_*a*_, *V*_*m*_, *K*_*m*_, *V*, *s*, *ω*_11_, *ω*_12_, *ω*_22_, *σ*) which gives a total number of 9 parameters to be estimated.

**Fig. 1:**
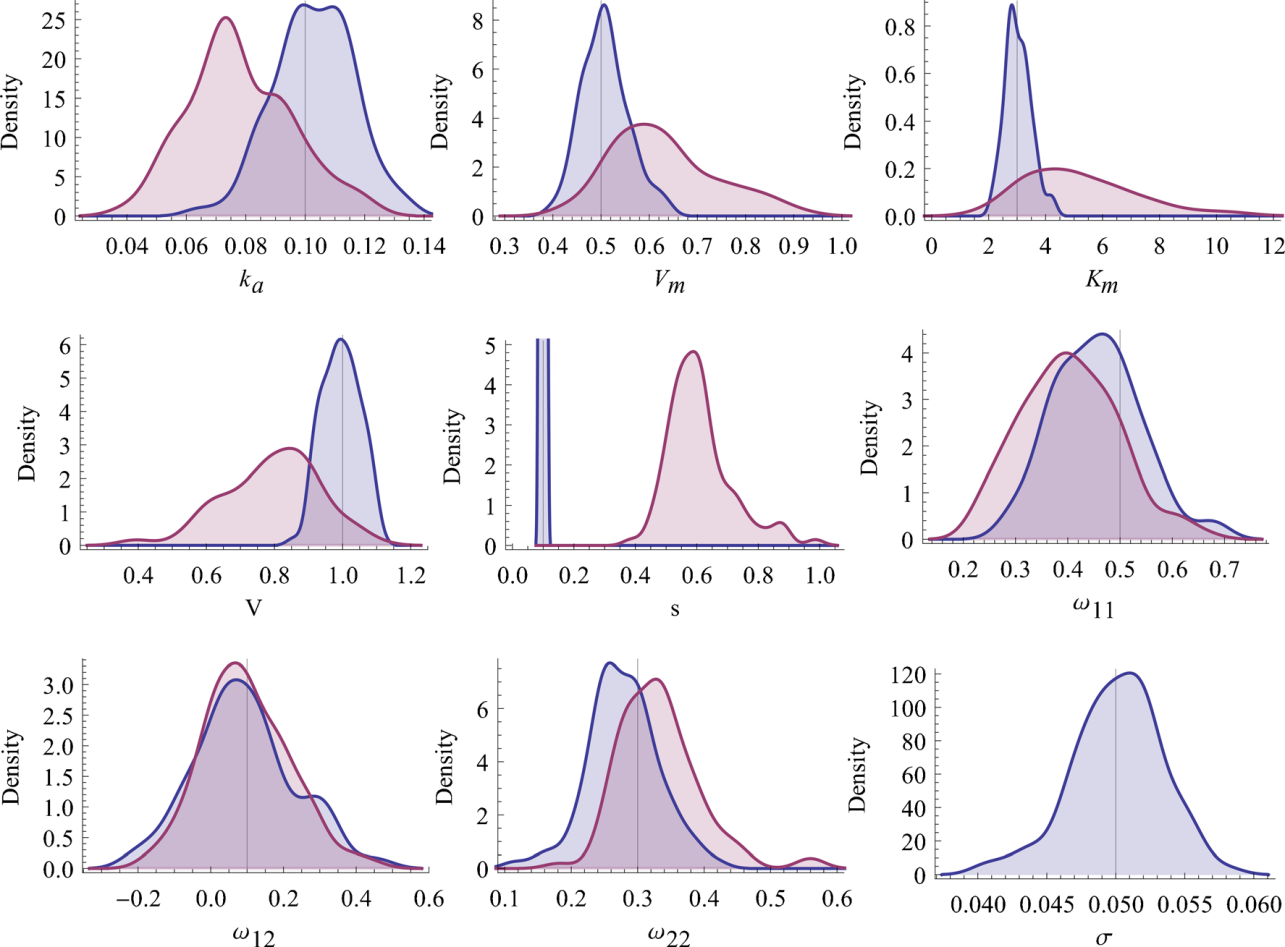
Smoothed histograms over the estimated parameters from 100 simulated data sets. The estimated parameters using the SDE model is shown in *blue* and the estimates using the ODE model (*σ* = 0) is shown in *purple. The vertical lines* show the parameter values used for simulation

We estimate the model parameters using the SDE approach and compare this to the corresponding ODE model (*σ* = 0). The estimated parameter values using the SDE model and the ODE model are shown in Table I. The relative standard errors (RSEs) in % are included in parentheses. They are obtained by calculating the mean and standard deviations of the 100 estimates. The distribution of the estimated model parameters for the 100 simulated data sets are seen in Fig. 1.

- Application 2: Stochastic NiAc disposition in obese Zucker rats

**Fig. 2:**
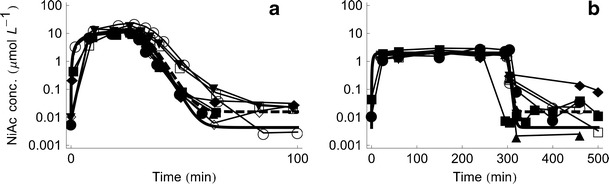
Plots of the estimated ODE (*solid*) and SDE (*dashed*) NiAc model together with the observed concentration time courses of NiAc for the two infusion groups. **a** 20 $\mu$mol kg^−1^ over 30 min. **b** 51 $\mu$mol kg^−1^ over 300 min over 300 min. The concentration is shown on a log-linear scale

The measured NiAc concentrations for the two infusion groups are shown in Fig. 2.

### Estimated Parameters

The parameters are estimated using the FOCE approximation of the individual Hessians. The reason for using the FOCE approximation is to guarantee that the individual Hessians are positive definite. The starting values for the structural parameters were adopted from Ahlström et al. (39) with values *V*_*m*_ = 1.8 $\mu$mol min^−1^ kg^−1^, *K*_*m*_ = 23 $\mu$mol L^−1^, *V*_*c*_ = 0.319 L kg^−1^, and *Synt* = 0.00125 $\mu$mol min^−1^ kg^−1^. Moreover, the starting values for the variance components were *s* = 0.1, *ω* = 0.1, and *σ* = 0.01 L min^−1^ (for the SDE model).

Table II shows the estimated parameters for the two models of interest. As a reference, we also provide the estimates from Ahlström *et al*. (39). The relative standard errors (RSEs) in % are included in parentheses, calculated from the approximated Hessian at the optimum. Two comparisons are of interest. First, we used the original NiAc disposition model (15) to compare the results from our estimation with the results from (39). Second, we are interested in the differences in parameter estimates using the original NiAc disposition model (15) and the stochastic NiAc disposition model (17).

**Table II:** Estimated parameter values and interindividual variability (IIV) for the NiAc disposition model, with corresponding relative standard errors (RSE %)

Parameter	Definition	Ahlström *et al*.	Current investigation: ODE model	Current investigation: SDE model
*V*_*m*_	Maximal velocity	1.59 (13.9)	1.46 (16.3)	1.35 (16.7)
*K*_*m*_	Michaelis-Menten const.	18.9 (21.5)	15.2 (21.7)	13.6 (21.5)
*V*_*c*_	Central volume	0.328 (12.4)	0.29 (4.3)	0.32 (5.5)
*Synt*	Endogenous synthesis rate	0.00280 (10.1)	0.0006 (29.5)	0.0018 (24.3)
*s*	Residual prop. error	0.400 (26.3)	0.460 (8.08)	0.241 (11.7)
*ω*	Variability *V*_*m*_	0.214 (234)	0.174 (22.5)	0.133 (27.0)
*σ*	System noise factor	-	-	0.033 (15.7)

See Ahlström *et al.* (39) for reference

### Fitted Population and Individual Models

The fitted population models for the two approaches are illustrated in Fig. 2.

**Fig. 3:**
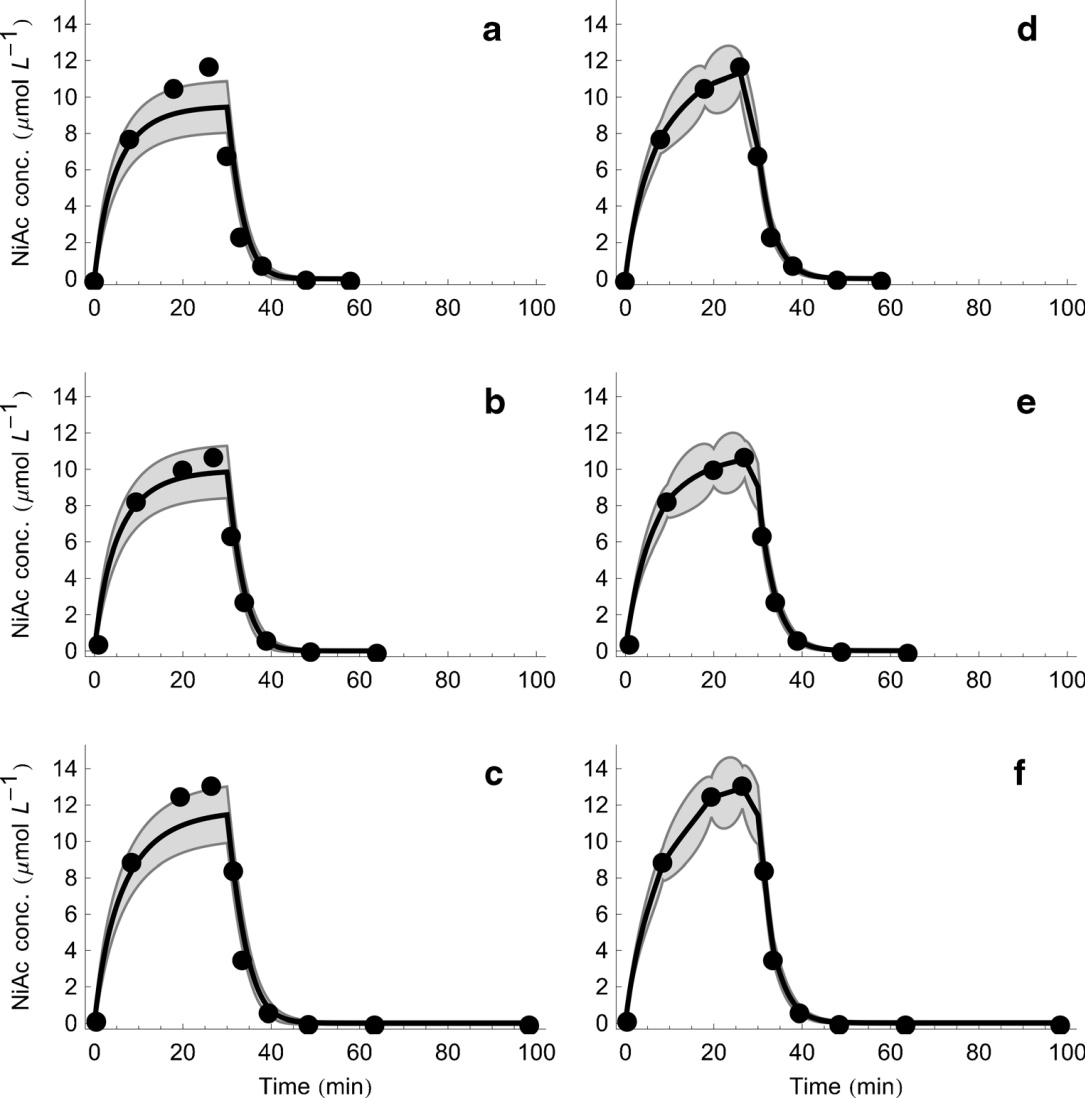
Observed plasma NiAc concentration time profiles together with the estimated ODE (**a–c**) and SDE (**d**–**f**) NiAc disposition model for three animals (*each row*) from the first infusion group (20 $\mu$mol kg^−1^ over 30 min)

Given the estimated population parameters as priors, the individual likelihoods are maximized once again to obtain the *maximum a posteriori* estimates for the random effect parameters. These optimal parameter values are then inserted in the model equations to obtain the individual model fits. The original NiAc disposition model fit is obtained by simply solving the ODE describing NiAc concentration given an individual’s parameter values. For the stochastic NiAc disposition model, the individual fit is slightly more complicated to obtain. Due to the stochastic component, the individual model fits for the stochastic model are obtained by a method called smoothing. This has previously been demonstrated by Kristensen *et al*. (2). When smoothing is used, the model is used to provide an optimal state estimator given all the measurements for a specific individual. The fitted individual models together with the output uncertainties are illustrated in Fig. 3 for three animals (rows 1–3) with the shorter infusion for the estimated ODE (a–c) and SDE (d–f) NiAc disposition model. The uncertainty bands represent one standard deviation of the output uncertainty (that is, the square root of the output covariance). Note that the concentration and its uncertainty are visualized on a linear scale, in contrast to the previous plots in Fig. 2, to emphasize the improved fit for large NiAc concentrations using SDEs.

## DISCUSSION

The extension of NLME models to stochastic differential mixed effect models has been considered to provide a more general model to describe the error between model prediction and observed data. By utilizing the stochastic setting, the total error is divided into measurement noise and model uncertainty. Together with the population variability induced in a mixed effect model, we are able to account for a total of three sources of variability.

### Maximum Likelihood Estimation

We have taken the maximum likelihood approach to parameter estimation by combining an approximation of the population likelihood together with an extended Kalman filter for state estimation. In contrast to the estimation method proposed in (10, 23–25), we have further developed the method by considering interaction between the output covariance and random effects, referred to as the FOCEI method (33). The interaction between output covariance and random effects occurs for example in ODE models with a proportional measurement noise and in stochastic models where the output covariance depends on the Kalman update, which in turn depends on the individual response.

The parameters were estimated using the gradient-based method BFGS (35). In many applications, the gradient of the objective function is approximated using finite differences. However, numerical ODE solvers with an adaptive step length are known to introduce quantification errors to the objective function, making it nonsmooth on small scales (42). To overcome such problems, we utilized the sensitivity equations when calculating the gradient in the inner and outer optimization problem. The sensitivity equations were obtained by differentiating the system of differential equations and the extended Kalman filter equations with respect to the parameters in the model. This has been previously demonstrated by Leander *et al*. (11) in the single individual case, and we are preparing a manuscript that concerns the mixed effects case.

The extension to SDEs comes with an increased computational burden. Due to the fact that we now consider equations describing the time evolution of both mean and covariance of a stochastic process, a larger system of ODEs has to be solved. The implementation of the numerical machinery for parameter estimation also becomes more challenging and requires advanced numerical techniques such as the EKF. The stochastic mixed effects modeling framework has been implemented in Mathematica 9. An executable version of the code may be received from the authors upon request.

- Application 1: Simulation and estimation in the simulated stochastic one-compartmental pharmacokinetic model

As a first application of stochastic mixed effects modeling, we used a stochastic one-compartmental pharmacokinetic model for a simulation study. The aim was to investigate whether the three sources of variability (measurement error, population variability, and model uncertainty) could be separated using the proposed maximum likelihood estimation.

From the parameter estimates for the SDE model in Table I and the smoothed histograms in Fig. 1, we conclude that all estimated parameter values are close to their true values used for simulation. The measurement error standard deviation *s* and the system noise factor *σ* are identical to the true values using the SDE approach. Also, the correlation between the random effects is close to the true value for the SDE model. However, the RSE for the random effect correlation is very high, implying that the correlation is difficult to reliably estimate. This may depend on the fact that only 20 individuals were included in each data set.

In the ODE case, we conclude that all the structural parameters are biased and differ significantly from the true parameter values used for simulating the data. Comparing the results to the ODE case where the system noise is neglected (*σ* = 0), we can see that the measurement error standard deviation *s* is increased six-fold. Hence, we conclude that when the system noise is set to zero (equivalent to the ODE case), the measurement error standard deviation is increased to account for that variability, which is not unexpected since we neglect the variability in dynamics. We also conclude that the RSEs for the SDE model are generally lower than the RSEs for the ODE model, although the RSEs for *ω*_12_ and *ω*_22_ are slightly higher in the SDE model.

Most importantly, we conclude that we can successfully distinguish the three sources of variability, which is a necessary condition of the extended framework to be of practical value.

- Application 2: Stochastic NiAc disposition in obese Zucker rats

Using the original NiAc disposition model, and comparing the results with previous work, we can conclude that most of the parameter values are similar. Our estimated values differ most in *K*_*m*_ and *Synt*. Note that the quotients *V*_*m*_/*K*_*m*_ are similar in the work by Ahlström *et al*. (39) and in the current investigation (0.084 and 0.096, respectively). This may imply that there are problems estimating the parameters *V*_*m*_ and *K*_*m*_, whereas the quotient as such is identifiable. One striking difference between our estimation and previous results is that the relative standard error for the parameter describing the interindividual variability (IIV) is significantly lower in our investigation. The IIV for *V*_*m*_ was 234% in (39), while it is reduced to 22.5% in our investigation. This is most likely because instead of using finite difference approximation of the gradient in the optimization problem, we utilize sensitivity equations, yielding a more robust calculation of the gradient, which also seems to influence the precision of parameter estimates.

If we turn to our investigation and compare the ODE and the SDE NiAc disposition models, we can see that the endogenous synthesis rate *Synt* is increased for the stochastic model, which is also seen in the population model fits in Fig. 2. Moreover, another difference in the parameter estimates is that the measurement error is much lower (two-fold) for the stochastic model whereas the system noise factor significantly differed from zero. This implies that the error that existed in the original model (measurement error) is now separated into two parts, namely the measurement error and the system noise. The interindividual variability for the maximal rate *V*_*m*_ is slightly decreased when the stochastic NiAc disposition model is used. Hence, some of the population variability in the maximal rate *V*_*m*_ seen in the original NiAc disposition model may instead be explained by a model uncertainty.

With respect to the individual fits in Fig. 3, there is a clear difference in terms of model fits. The original NiAc disposition model, proposed in (39), seems to underestimate the drug concentration during the infusion. The stochastic model that we propose is much closer to the measurements and seems to account for model deviation that the original model is not capable of. In the original NiAc disposition model, the output covariance is equal to the measurement covariance, whereas in the stochastic model, it is a combination of the state covariance and measurement covariance.

That means that the confidence band in the original model simply arises from the variance of measurements, which we assumed to be proportional to the concentration level. Using the stochastic model, we conclude that the uncertainty is highest between two consecutive time points. In contrast to the original model, we have a decreased uncertainty at the measurements, at which information is gained about the underlying system.

In previous work (39), the original NiAc disposition model was used to drive a pharmacodynamic model describing production of NEFA. By utilizing the stochastic NiAc disposition model, the fitted individual models seem to be able to capture the high concentrations during the infusion.

This is seen in Fig. 3 and may give a better input to the NEFA model. A better input to a pharmacodynamic model can be of broader interest in PKPD modeling, since a deterministic pharmacokinetic model often is used to drive a pharmacodynamic model. Using a stochastic pharmacokinetic model can better account for uncertainty in the drug kinetics.

## CONCLUSIONS

We conclude that the stochastic modeling framework we proposed here leads to a more general framework for handling measurement error and model errors. This framework, together with an effective method for calculating the gradients in the nested optimization problem, provides us with a flexible, robust modeling framework for mixed effects models.
